# Multi-omics reveals the effects of dietary supplementation with *Bupleuri radix* branch powder on gut microbiota and lipid metabolism: insights into gut microbial-muscle interactions

**DOI:** 10.1128/spectrum.01457-24

**Published:** 2024-10-22

**Authors:** Haiyan Li, Cheng Pan, Fuqiang Wang, Zengkai Li, Khuram Shahzad, Yanping Huang, Wangsheng Zhao

**Affiliations:** 1 School of Life Sciences and Engineering, Southwest University of Science and Technology, Mianyang, Sichuan, China; 2 Shenmu Livestock Development Center, Yulin, China; 3 Department of Biosciences, COMSATS University Islamabad, Islamabad, Pakistan; National Institutes of Health, Bethesda, Maryland, USA

**Keywords:** *Bupleuri radix* branches powder, Shaanxi fine-wool sheep, growth and immune performance, multi-omics

## Abstract

Improving livestock growth and raising the quality of livestock products have attracted much attention owing to the market’s growing need for livestock products. *Bupleuri Radix* branches powder (BR) has a variety of health characteristics, but its effects on ruminant growth and animal product quality are still uncertain. This study explored the effects of BR on growth performance, health status, gut microbiota, and muscle lipid metabolism of Shaanxi fine-wool sheep (SFS), and examined the interaction between gut microbiota and lipid metabolism through correlation analysis. The results indicated that BR can regulate the immune function, intestinal VFAs, and enzyme activity of FSF by improving the gut microbiota, thereby affecting its muscle lipid metabolism. The lipid metabolite TG showed a strong positive correlation with the gut microbes *Bacteroides* and *Fibrobacter*, while Phosphatidylethanolamine and Phosphatidyl serine (PE and PS) showed a significant negative correlation with *Fibrobacter*. The above results indicate that gut microbiota and lipid metabolites interact with each other. BR has the effects of promoting SFS growth, improving body health, and improving meat quality. These findings offer new insights into improve animal growth performance and livestock product quality in modern farming.

**IMPORTANCE:**

Enhancing livestock growth performance and improving meat quality are important guidelines for the development of the current animal husbandry industry; thus, we explored a comprehensive study of *Bupleuri Radix* (BR) on growth performance, gut microbiology, and muscle lipid metabolism in Shaanxi fine-wool sheep (SFS). Our research has found that BR could improve the growth performance of SFS and meat quality by affecting gut microbes. This study provides new solutions to improve the economic efficiency of animal husbandry.

## INTRODUCTION

Mutton is popular among customers due to its low fat, cholesterol, and high protein content. China is one of the world’s largest producers of mutton and occupies a pivotal position in the production of sheep meat for animal husbandry. The market’s demand for mutton has gradually grown as living conditions have improved, and mutton quality has also become more demanding (1). Shaanxi fine-wool sheep (SFS) is a dual-purpose breed, raised for both meat and wool in northern Shaanxi for more than 30 years. Its traits include good feed intake, resilience to cold and drought, and strong adaptability. However, its adult sheep’s subcutaneous fat is thick, smell of mutton, and experiences delayed growth and a low feed payoff rate, among other issues (2). Investigating ways to enhance SFS’s growth performance and the quality of lamb meat is therefore crucial from both economic and social significance.

Mostly composed of saponins, flavonoids, essential oils, alkaloids, and polysaccharides, *Bupleuri radix* (BR) is a common Chinese herbal (3). The pharmacologically active ingredients of BR, a by-product of *Bupleuri radix*, are comparable to those of *Bupleuri radix* and have the same anti-inflammatory, antiviral, antioxidant, and immunomodulatory properties (4–6). A growing number of research has demonstrated that BR is a beneficial feed supplement that also improves meat quality and animal growth. Cheng et al. (7) found that BR can be used as a useful feed additive. The addition of BR to the diet can improve the specific growth rate and feed conversion rate of white shrimp (*Litopenaeus vannamei*). Angela et al. (8) discovered that BR decoction enhances grouper growth and antioxidant capacity, as well as liver lipid production and the prevention of disorders related to the liver. Zou et al. (9) added *Bupleuri radix* extract to the diet and found that *Bupleuri radix* improved immunity and antioxidant capacity in broiler chickens and increased growth performance and meat yield. However, the effects of BR on ruminant growth, immunological function, and meat quality have not been thoroughly studied, and it is still unknown what mechanisms are underlying BR’s regulation of SFS growth, immune function, and meat quality.

The greatest immune system in an animal is found in the gut, which is also a crucial location for digestion and absorption. The gut microbes interact with the host in a symbiotic, coevolved, or parasitic relationship; it influences the host’s physiology, immunity, health, and productive capacity and is crucial for immunoregulation, growth, and development (10–13). Research has demonstrated that, in addition to its capacity to regulate intestinal bacteria and have a repairing effect on intestinal damage, *Bupleuri radix* can be used to treat gastrointestinal diseases with little to no adverse effects (14, 15). Intestinal bacteria fermentation and metabolites contribute to lipid synthesis and metabolism, which can lower fat deposition and have a direct or indirect impact on the flavor, taste, and quality characteristics of meat (16, 17). For instance, there are physiological mechanisms in which an increase in the number of *lactobacillus* in the colon and a decrease in the number of *Escherichia coli* not only have a positive regulatory effect on backfat thickness and carcass weight but also significantly improve the overall quality of pork (18). According to relevant studies, the concomitant rise in beneficial intestinal bacteria in lambs can influence the development of their skeletal muscles by controlling the gut-muscle axis (19). It can also enhance the quality and quantity of the muscles by reducing shear force and increasing myofiber diameter and cross-sectional area (20). The previous study indicates that *Bupleuri radix* may enhance gut microbes, and meat quality is strongly correlated with gut bacteria and associated metabolites (7, 9). Whether adding BR to the diet may effectively control the intestinal bacteria and enhance the quality of SFS meat is still unknown.

Hence, the core objective of this study is to analyze the effects of BR on SFS growth and immune performance, gut microbiota, and muscle lipid metabolism, and to explore the interaction between gut microbiota and muscle lipid metabolism. The results showed that dietary supplementation of BR could promote the growth and immune function of SFS. Concurrently, BR can improve meat quality by regulating gut microbiota and muscle lipid metabolism. This finding provides a new nutritional pathway for improving the growth performance and meat quality of SFS and provides new enlightenment.

## MATERIALS AND METHODS

### Experimental design and sample collection

The SFS for the experiment were provided by the SFS original breeding farm in Shenmu City, Shaanxi Province. Forty SFS (half male and half female) that had been weaned at the age of 3 months, with similar body weight and robustness, were selected for the experiment, and then each of the selected SFS was divided into pens and fed under 3 m^2^ conditions, and they were randomly divided into four groups of 50% females and 50% males (*n* = 10). The groups were treated as follows: the control group (CON) was fed the basal diet; the treatment group was supplemented with BR (BR1 %, BR 2%, and BR4 %) in the diet at 10 g/kg, 20 g/kg, and 40 g/kg, respectively. The basal diet and nutritional levels of SFS during the experiment were referred to by Pan et al. (4) (Table 1). The total test period was 67 days, comprising a 1-week pre-test and a 2-month main test period. BR bought from the Chinese herbal medicine sales company “Shixuantang.” The SFS body weight, body length, height, chest size, and shin circumference were measured at the beginning and end of the experiment used for a comprehensive assessment of their growth performance. Blood was collected *via* jugular vein sampling and centrifuged at 3,500 r/min for 15 minutes at a constant temperature of 4°C to efficiently separate the serum, which was then dispensed into cryopreservation tubes and subsequently stored in liquid nitrogen for the determination of serum hormone levels and immunological parameters. After the experiment, stool samples for the test were collected using rectal feces, dispensed into 2 mL cryopreservation tubes, and stored in liquid nitrogen for subsequent analysis of intestinal enzyme activity, volatile fatty acid (VFAs) assay, and DNA extraction from the gut microbiota. At the end of the study, based on the health status and growth performance of SFS, the CON group and the group with the best growth performance (BR4) were slaughtered and the longest dorsal machine was collected from both groups. The collected muscles were stored in liquid nitrogen in portions for subsequent use in lipid metabolism assays, while the other portion was extracted as 3 cm^2^ sized pieces of muscle tissue in 50 mL centrifuge tubes containing 4% paraformaldehyde and kept at 4°C. To ensure the quality and stability of the samples, the collected samples were stored at the required temperature after they were brought back to the laboratory.

**TABLE 1:** Basic diet composition and nutrient levels (4)

Ingredients	Content
Corn	28.85
Soybean meal	7.50
Premix	3.20
Alfalfa grass	22.45
Corn for silage	38.00
Total	100
Nutrient levels (%)	
Crude protein	18.91
Crude fat	1.80
Crude fiber	10.79
Crude ash	3.70
Ca	0.32
P	0.15
Lysine	≥0.4
Digested energy	11.13%

### Determination of serum hormones and immune indexes

To assess the hormone concentrations and immunological indices in the serum samples, the collected serum samples were thawed at 4°C, and the relevant indices were determined by the Elisa method using commercial assay kits provided by Nanjing Jiancheng Bioengineering Institute (Nanjing, China). The serum hormone indices measured mainly include growth hormone (GH), insulin (INS), insulin growth factor-1 (IGF-1), and thyroxine 4 (T4), which are key hormones closely related to growth. In addition, parameters of key immune indicators such as immunoglobulin A (IgA), immunoglobulin G (IgG), immunoglobulin M (IgM), interleukin-6 (IL-6), and tumor necrosis factor-alpha (TNF-α) were also measured.

### Determination of intestinal enzyme activity and VFA acids

Collected fecal samples were thawed at 4°C and intestinal enzyme activities such as cellulase, pepsin, lipase, and amylase were assessed using the Elisa assay according to the assay provided in the commercial assay kits supplied by Nanjing Jiancheng Bioengineering Institute (Nanjing, China). To determine the information on the content of VFAs such as acetic, propionic, butyric, valeric, and isovaleric in fecal, 1 g of thawed fecal sample at 4°C was weighed and transferred to an appropriate centrifuge tube. Add 2 mL of 0.1% hydrochloric acid, mix immediately, ice bath for 25 min, then centrifuge for 15 min at 15,000 rpm at 4°C constant temperature. The supernatant was withdrawn using a syringe and passed through a 0.22-µm filter membrane (Millipore, USA), filtered to remove impurities and residues into a sample vial, and injected into a gas chromatograph (Agilent HP 6890 series, USA) for determination of VFAs.

### Production and observation of tissue sections

After being removed from the 4% paraformaldehyde-fixed tissue, it was rinsed and dehydrated using 75% alcohol for 4 h, 85% alcohol for 2 h, 95% alcohol for 1 hour, and 100% alcohol for 5 h. After the procedure was carried out four times, the tissue was dehydrated, soaked in xylene for 10 minutes to make it transparent, embedded in paraffin wax, and sectioned once the paraffin wax fixation was finished, followed by staining with hematoxylin and eosin. All of the specimens were then sealed with neutral gum. Every specimen was handled in accordance with the pathological examination standard operating procedure. The prepared tissue sections were examined through a microscope with multiple magnifications, and the images were captured.

### Microbiome sample processing and sequencing

The Tian Gen Fecal Genomic DNA Extraction Kit is designed to efficiently extract genomic DNA from fecal samples using the CTAB method, which is evaluated on a 1% agarose gel to ensure satisfactory quality and concentration of the extracted DNA. Subsequently, based on the assessed DNA concentration, an appropriate amount of DNA was precisely extracted in a centrifuge tube and diluted to a concentration of 1 ng/µL in sterile water for subsequent PCR amplification. The PCR system consisted of 15 µL of Phusion Master Mix (2×), 0.2 µL each of upstream and downstream primers, and 10 µL of the diluted 1 ng/µL DNA sample, ddH_2_O 4.6 µL to make the PCR system 30 µL. The PCR amplification program was as follows: pre-denaturation at 98°C for 1 min; 30 cycles including (denaturation at 98°C for 10 s; annealing at 50°C for 30 s; extension at 72°C for 30 s); and finally extension at 72°C for 5 min. PCR products were mixed in equal amounts with 1× buffer containing fluorescence and purified by agarose gel electrophoresis at a concentration of 2%, followed by recovery of the products using the Qiagen Gel Extraction Kit (Qiagen, Germany). Sequencing libraries were created using the TruSeq DNA PCR-Free Sample Preparation Kit, and the constructed libraries were evaluated for quality using Qubit and Agilent Bioanalyzer 2100, and quantified by System/Q-PCR; after the libraries were qualified, the libraries were sequenced using the Illumina. After the libraries were qualified, sequencing was performed using the Illumina NovaSeq 6000.

### Bioinformatics and statistical analysis of bacterial metagenomics

Raw data were filtered, spliced, and compared to obtain valid data using fastp (v0.22.0), FLASH (v1.2.11), and vsearch (v2.22.1). Primer sequences were trimmed and chimeric sequences were removed using cutadapt 1.9.1 and UCHIME v4.2 software. Using Quantitative Observations in Microbial Ecology (QIIME) software (Uparse v7.0.1001), all sequences and microbiota compositions were classified and clustered into OTUs with 97% similarity of the sample sequences to calculate alpha diversity. Alpha diversity was analyzed in depth using the R program (v3.6.0) to explore species abundance and evenness and to verify that the current sequencing depth is sufficient to cover the vast majority of species information. Intergroup differences (phylum and genus) at the species taxonomic level were analyzed using Mothur software to assess changes in microbial composition membership and differences in a relative abundance of microbiota.

### Muscle sample treatment and lipid metabolite analysis

The metabolomic profiling of muscle lipids has been comprehensively analyzed using liquid chromatography-tandem mass spectrometry (LC-MS/MS). The collected muscle samples were ground (about 20 mg) for muscle lipid metabolite extraction using a ball mill (TissueLyser; Qiagen, Beijing, China) under liquid nitrogen conditions. The muscle lipid extraction process consists of first adding 1 mL of lipid extract to the muscle tissue, vortexed for 15 min to mix the sample and extract, then adding 200 µL of water, vortexed for 1 minute, and centrifuging at 12,000 r/min for 10 min at 4°C to separate the sample mixture. The supernatant was then transferred to a centrifuge tube and concentrated until completely dry. Add 200 µL of lipid re-solution, vortex for 3 min, and centrifuge at 12,000 r/min for 3 min.

The data acquisition instrumentation system mainly included ultra-performance liquid chromatography (UPLC) and tandem mass spectrometry (MS/MS), and the liquid phase conditions mainly included: chromatography column (Thermo Accucore C30 column, i.d. 2.1 × 100 mm, 2.6 um), mobile phase A: 60% acetonitrile aqueous solution (containing 0.1% formic acid, 10 mmol/L formic acid); mobile phase B: 10% acetonitrile isopropanol solution (containing 0.1% formic acid, 10 mmol/L formic acid); flow rate of 0.35 mL/min; column temperature of 45°C; and injection volume 2 µL. The mass spectrometry conditions mainly included: electrospray ionization (ESI) temperature 500°C, mass spectrometry voltage 5,500 V in positive ion mode, mass spectrometry voltage −4,500 V in negative ion mode, ion source gas1 (GS1) 45 psi, gas2 (GS2) 55 psi, and curtain gas (CUR) 35 psi. The collision-activated dissociation (CAD) parameter was set to Medium. In the triple quadrupole, each ion pair was scanned for detection based on optimized declustering potential (DP) and collision energy (CE). The software was made to fill in the missing values with the k-nearest neighbor algorithm (KNN 1.56.0), and then the QC sample CV values were calculated, and substances with CV values less than 0.3 were retained to obtain the final data. After analyzing the data, principal component analysis (PCA) and orthogonal partial least squares discriminant analysis (OPLS-DA) were performed to show between-group differences in metabolic profiles. For two-group analyses, differential metabolites were identified by *P*-values < 0.05, variable effects on projection and (VIP) >1 were used to determine differential metabolites.

### Data analysis

SPSS (v21.0) software was used for statistical analysis. We used Origin (2021) software for plotting. In this software, multiple group comparisons were analyzed using ANOVA, and two-by-two comparisons were made using a *t*-test. *P* < 0.05 was considered to be a significant difference, and *P* < 0.01 was considered to be a very significant difference.

### Correlation analysis

Spearman was used to analyze the correlation test. The Spearman correlation coefficient was calculated using the cor function of R software, the significance test was performed using the corPvalueStudent function of R software, and the drawing was performed using the ComplexHeatmap of R software.

## RESULTS

### Effects of BR on the growth performance of SFS

The growth performance of SFS was ascertained to comprehend the impact of BR on the performance. As can be seen in Table 2, SFS’s initial body weight did not differ significantly (*P* > 0.05), and the supplemented with BR to the diet did not affect the SFS of body length, height, chest size, or shin circumference significantly. Following the final stage of the experiment, significant increases in final body weight and mean daily weight gain were observed in the BR2 and BR4 groups compared to the CON group, with significant advantages (*P* < 0.05), and the final weight of the BR4 group was as high as 42,85 kg and the average daily gain was as high as 0.3 kg (*P* < 0.05) compared to CON group. Therefore, it can be hypothesized that BR has a positive effect on the growth performance of SFS, with BR4 having the best conditional benefit.

**TABLE 2:** Effect of BR supplementation on SFS growth performance^*a*^

Items	CON	BR 1%	BR 2%	BR 4%
Starting weight (kg）	24.85 ± 3.65	24.94 ± 3.98	24.74 ± 4.94	24.61± 5.17
Final weight (kg）	35.09 ± 4.54^a^	36.84 ± 5.96^a^	39.01 ± 4.77^ab^	42.85 ± 4.90^b^
Average daily weight (kg）	0.17 ± 0.03^a^	0.20±0.04^a^	0.24 ± 0.03^b^	0.30 ± 0.03^c^
Body length (cm）	65.70 ± 3.98	69.30 ± 6.55	67.10 ± 5.47	69.80 ± 3.99
Height (cm）	63.50 ± 3.46	66.20 ± 3.49	66.20 ± 5.81	64.50 ± 3.21
Chest size (cm）	88.90 ± 8.16	88.70 ± 8.92	87.10 ± 10.66	90.80 ± 7.04
Shin circumference (cm）	11.00 ± 0.67	10.85 ± 1.11	10.55 ± 1.12	11.00 ± 1.05

^*a*^ Note : Different letters ( a, b, c ) indicate statistical significance. Shoulder labels in peer data that did not contain the same letter indicated significant differences between these data *P* < 0.05, there was no significant difference between the same letters or no letters *P* > 0.05).

### Effects of BR on immune markers and hormones in SFS

**Fig 1:**
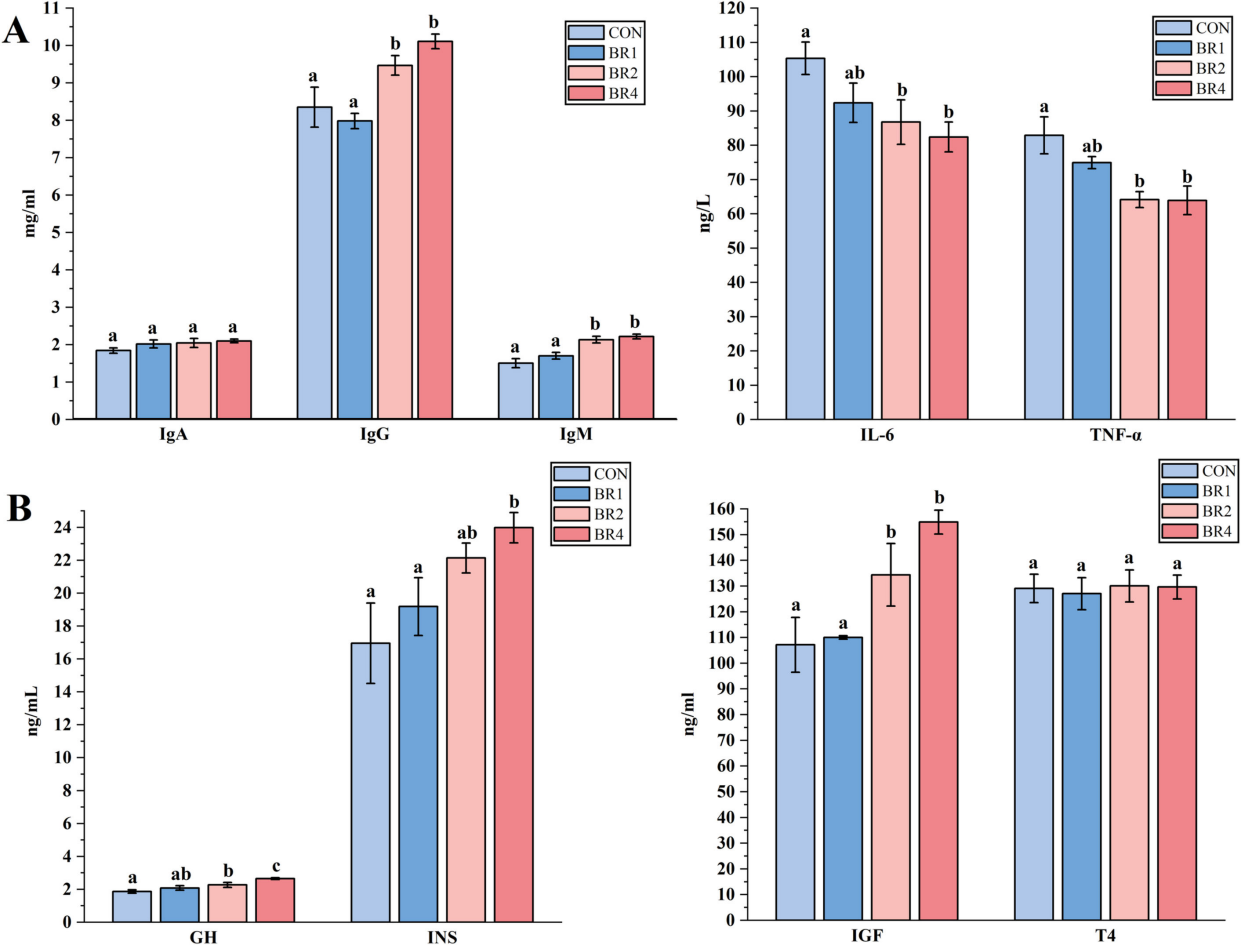
Effects of BR on immune markers and hormones in SFS (Note : (A) immune index; (B) Hormone levels. The error bar does not contain the same letter indicates a significant difference *P* < 0.05, with the same letter means no significant difference *P* > 0.05）.

Targeted testing of SFS serum immune markers and hormone levels to gain greater insight into the potential impact of BR on SFS health status, growth, and immune performance. In this research, serum IgG, IgM, GH, INS, and IGF levels were significantly higher and IL-6 and TNF-α levels were significantly lower in the BR2 and BR4 groups than in the CON group (*P* < 0.05), and the highest levels of IgG, IgM, GH, INS, and IGF were found in the BR4 group, in contrast, IL-6 and TNF-α levels in the BR4 group had the levels were the lowest in the BR4 group (Fig. 1A and B). Based on the aforementioned data, it is possible to speculated that BR can enhance SFS’s growth, immune system function, and general health, with the BR4 group showing the greatest results.

### Effects of BR on VFAs and enzyme activities in the gut of SFS

**Fig 2:**
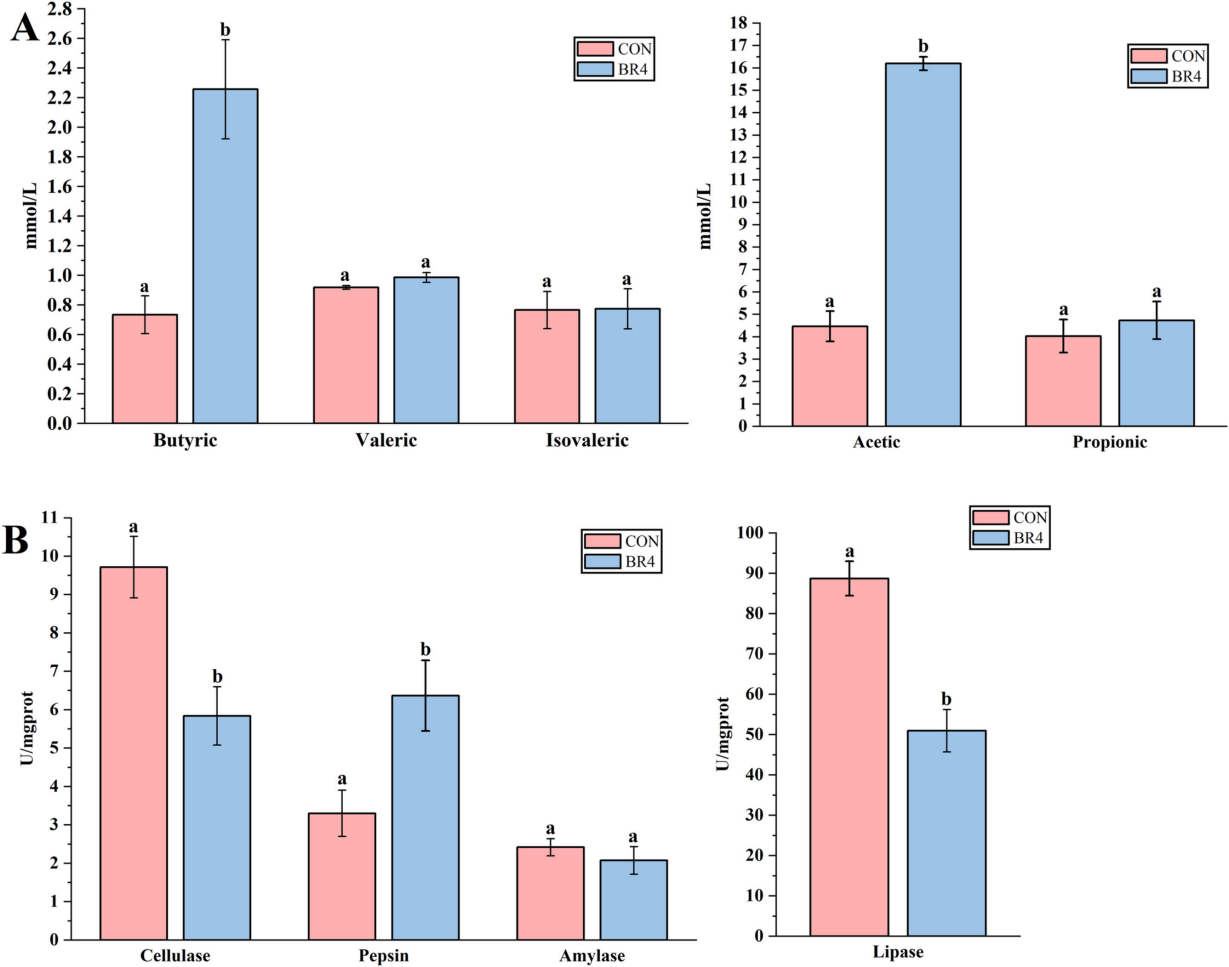
Effects of BR on VFAs and enzyme activities in the gut of SFS (Note: (A) intestinal VFAs; (B) intestinal enzyme activity, error bar does not contain the same letter indicates a significant difference *P* < 0.05, with the letter means no significant difference *P* > 0.05).

Due to the BR4 group’s better development and immunological function, intestinal VFAs and enzyme activities were found to be highly significant in both the regulation of meat quality and nutrient digestion and absorption. As demonstrated in Fig. 2, the concentration of VFAs such as butyric and acetic as well as pepsin activity were significantly higher in the BR4 group compared to the CON group *(P* < 0.05*)*, while the BR4 group’s intestinal activities for lipase and cellulase were significantly lower than those of the CON group *(P* < 0.05). Concentrations of VFAs such as propionic, valeric, and isovaleric as well as α-amylase activity were not significantly different between the two groups (*P* > 0.05). These findings lead to the hypothesis that BR may enhance SFS’s gut health and nutrition absorption.

### Effects of BR on gut microbiota of SFS

**Fig 3:**
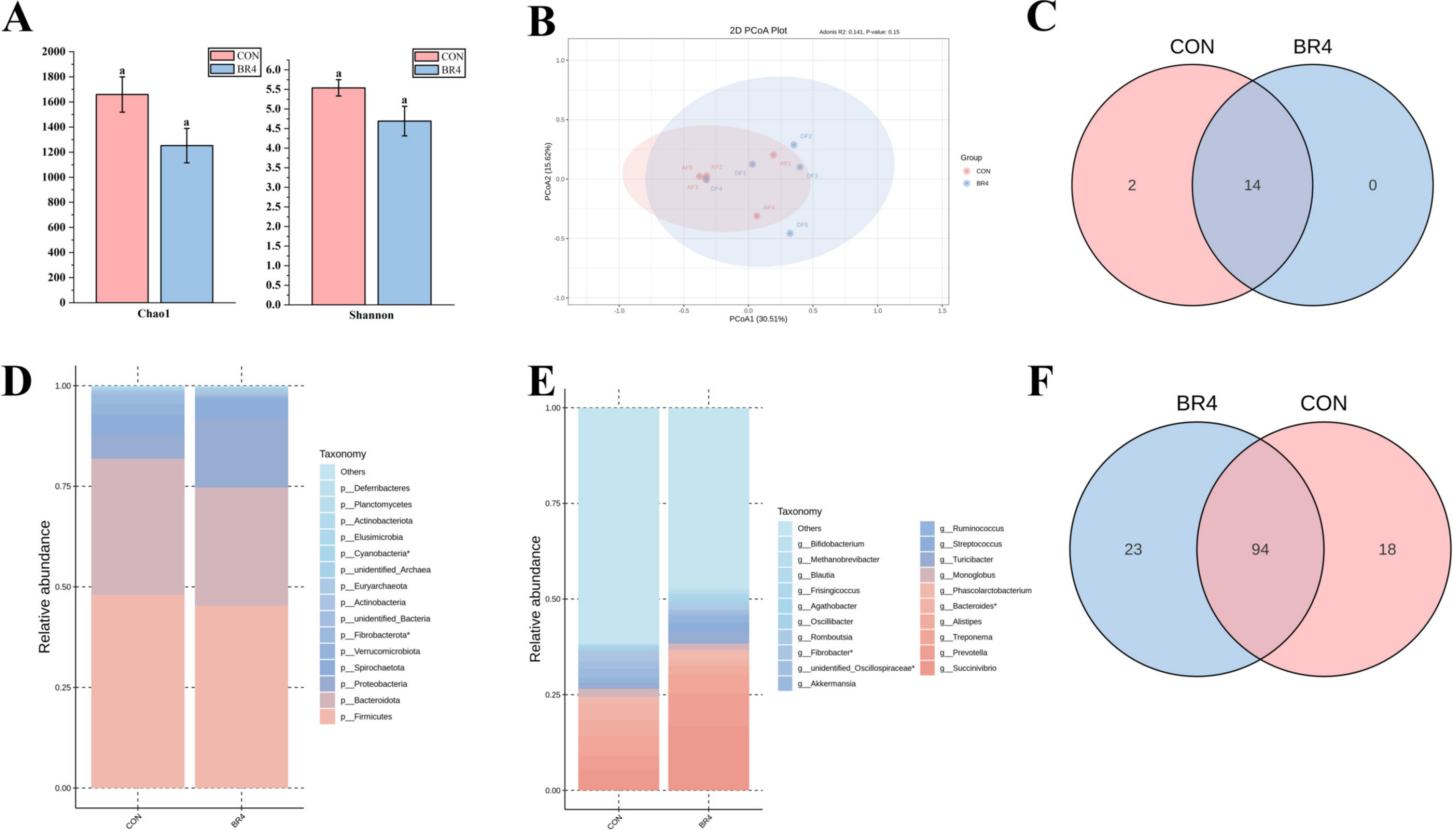
Effect of BR on intestinal microorganisms in FSF (Note: (A) Shanno and Chao1 indices; (B) PCoA results; (C) phylum level on the Venn diagram; (D) changes in gut microbiota composition at the phylum level; (E) changes in gut microbiota composition at genus level; and (F) genus gate level on the Venn diagram).

To further explore the effect of BR on gut health, the gut microbiota was compared and analyzed. After all test samples were sequenced and examined, primers were used to amplify the V3 and V4 regions, and the sequences obtained were then scrutinized according to the 97% similarity criterion and OTU grouping analysis. The results of α-diversity analysis showed that there was no significant difference in Shannon index and Chao1 index between the CON and BR4 groups. It can be surmised that BR supplementation in the diet had little effect on the abundance and diversity of gut microbiota (Fig. 3A). Based on the PCoA data (Fig. 3B), it can be inferred that feeding BR has a correlative effect on gut microbiota diversity since there was clustering of gut microorganisms in the BR4 and CON groups. This suggests that there was a fluctuation in *β*-diversity between the BR4 and CON groups. OTU clustering and taxonomy were used to examine changes in the phylum- and genus-level classification and abundance of microbiota. The Venn diagram (Fig. 3C) results indicate that, at the phylum level, the BR4 and CON groups shared 14 OTUs, or 87.5% of the total, whereas the CON group had two unique OTUs, or 12.5% of the total, and the BR4 group had no unique OUTs. At the genus level, the CON and BR4 groups share 94 OTUs, or 69.6% of the total, with 23 OTUs, or 17% of the total, exclusive to the CON group and 18 OTUs, or 13.4% of the total, exclusive to the BR4 group (Fig. 3F). At the phylum level, Firmicutes and Bacteroidota were the most dominant groups. Proteobacteria abundance increased by 11.5% in the BR4 group compared to the CON group, and Fibrobacterota and Cyanobacteria showed significant differences (*P* < 0.05), the relative abundance of Firmicutes and Bacteroidota decreased by 2.7% and 4.5%, respectively (Fig. 3D). *Succinivibrio*, *Prevotella*, and *Treponema* had the highest relative abundances of the BR4 group at the genus level, accounting for 16.5%, 8.7%, and 4.8% of the colony abundance, respectively, while *Bacteroides*, *Succinivibrio*, and *Treponema* accounted for 5.1%, 5.1%, and 4.2% of the colony abundance in the CON group, excluding miscellaneous bacteria. The BR4 group showed a 2.8% decrease in *Bacteroides* abundance an 11% rise in *Succinivibrio* abundance, and a 5.3% increase in *Prevotella* abundance when compared to the CON group. Notably, there were significant differences (*P* < 0.05) between *Oscillospiraceae* and *Bacteroides* (Fig. 3E). This implies that the variety and compositional structure of the SFS gut bacteria were impacted by the addition of BR to the diet.

**Fig 4:**
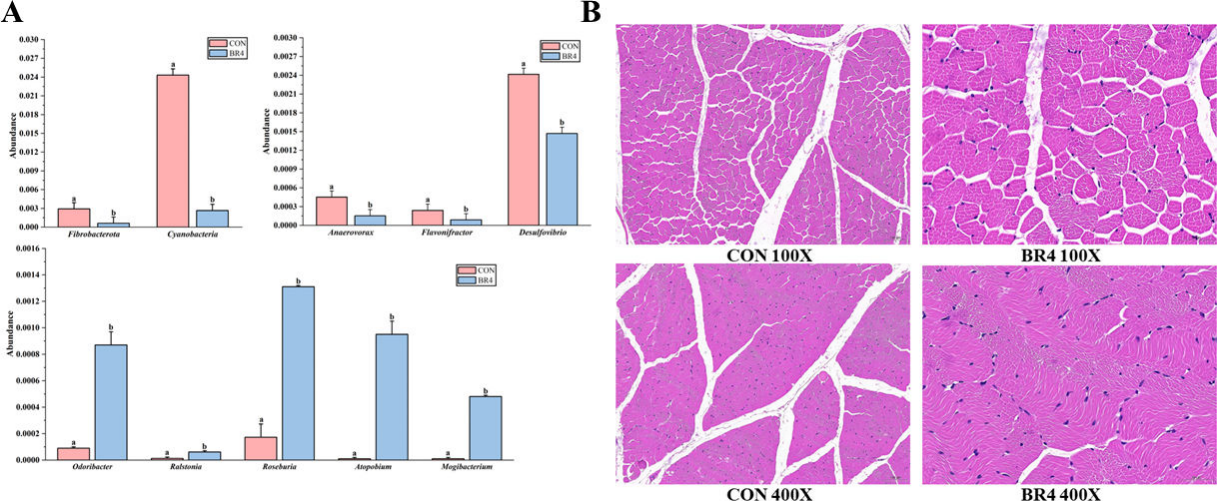
Intestinal significantly different microbiota and muscle tissue sections (Note: (A) intestinal differential microbiota and (B) muscle tissue sections).

Therefore, we performed differential relative abundance analyses using Metastat to examine changes in significantly different microbiota in the two groups to better understand the differences in relative abundance of the gut microbiota at the phylum and genus levels between the CON and BR4 groups. Figure 4A illustrates that, compared with the CON group, the BR4 group’s phylum level abundance of Fibrobacterota and Cyanobacteria was significantly lower (*P* < 0.05) than that of the CON group; conversely, the BR4 group’s genus-level abundance of *Flavonifractor*, *Anaerovorax*, and *Desulfovibrio* colonies was significantly lower (*P* < 0.05), while the abundance of *Ralstonia*, *Mogibacterium*, *Roseburia*, *Atopobium*, and *Oscillospira* was significantly higher (*P* < 0.05) (Fig. 4C). In the current study, findings showed that the gut microbiota interacted, with the addition of BR, the good gut microbiota were increased while preventing the growth and reproduction of the bad bacteria.

### Effects of BR on lipid metabolism in SFS muscle

**Fig 5:**
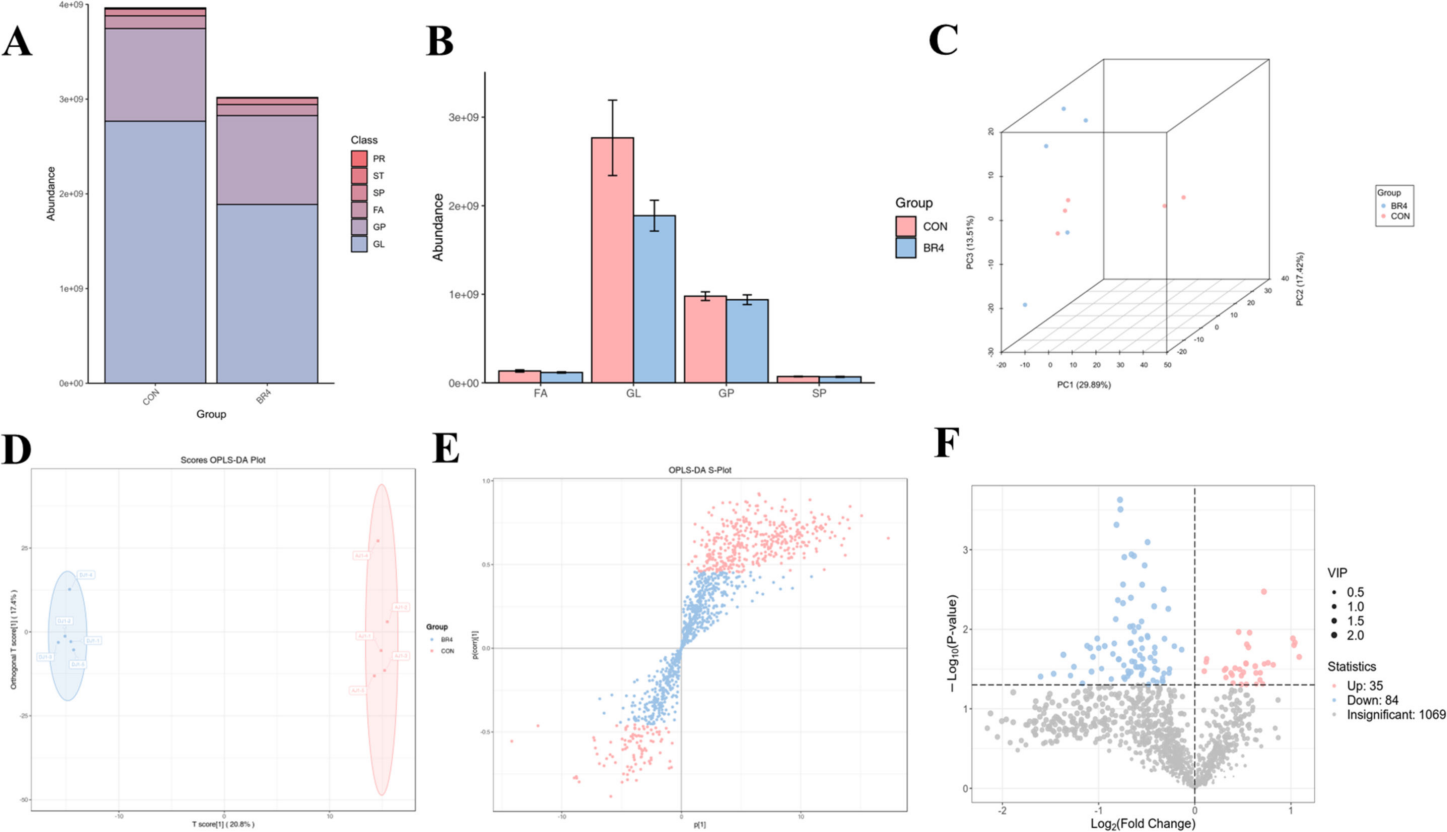
Effects of BR on lipid metabolism in SFS muscle: (A) lipid abundance analysis diagram; (B) relative lipid content diagram; (C) principal component analysis diagram; (D) OPLS-DA score map; (E) S-plot diagram of OPLS-DA; and (F) volcano plot.

We tested the meat quality using tissue sectioning and lipid metabolism to assess whether the addition of BR to the diet had any influence on SFS meat quality. The tissue section results demonstrated that, in contrast to the CON group, the BR4 group’s muscle fibers were small and dense, and that, concurrently, the structure of the muscle bundles was more clearly defined. The muscle tissue of the CON and BR4 groups also had thinner epimysium membrane without obvious thickening, and the muscle bundles had clear structure and well-defined boundaries without myocyte atrophy, hypertrophy, or necrosis (Fig. 4B). We identified 1,188 distinct lipid metabolites, which were divided into four groups viz, glycerolipids (GL), glyceryl phospholipid (GP), fatty acids (FA), and sphingolipids (SP). We used LC-MS/MS technology to anticipate and analyze the pertinent activities of muscle lipids. Glycerol lipid (GL) content was relatively low in the BR4 group compared to the CON group, and there were no appreciable variations in the total amounts of glycerol GL, GP, FA, and SP (Fig. 5A and B). The lipid metabolites of the CON and BR4 groups were found to be clustered separately in the principal component analysis (PCA) results (Fig. 5C). By contrast, orthogonal partial least squares discriminant analysis showed significant between-group differences in lipid metabolites between the two groups (Fig. 5D) and the appearance of significant differential lipid metabolites (Fig. 5E), indicating that the inclusion of BR in the diets may have had an impact on the lipid metabolism of SFS muscle.

**Fig 6:**
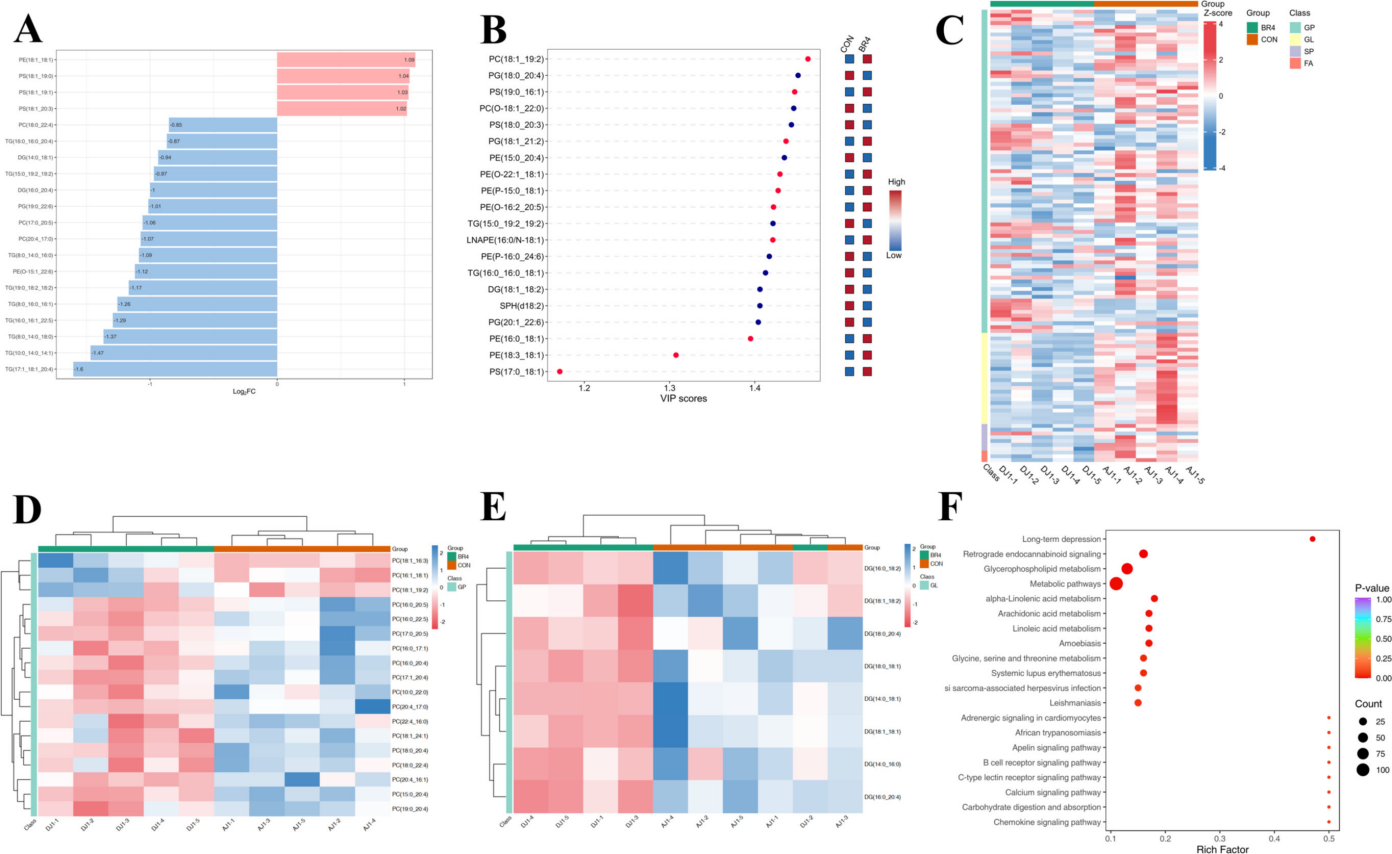
Differential lipid analysis: (A) differential lipid bar chart; (B) difference lipid VIP map; (C) differential lipid clustering heat map; (D) KEGG pathway GP differential lipid molecule clustering heat map; (E) KEGG pathway GL differential lipid molecule clustering heat map; and (F) differential lipid KEGG enrichment map).

We further investigated differences in lipid metabolites to gain insight into changes in muscle lipids in the two groups. As the volcano plot illustrates, we discovered that out of the 1188 lipid metabolites, 35 were significantly upregulated and 84 were significantly downregulated (Fig. 5F). Four lipid molecules were discovered to be upregulated and 16 to be downregulated when the top 20 lipid molecules with the multiplicity of difference between the two groups were selected for comparison (Fig. 6A). Compared with the CON group, 50% of the lipid metabolites in the BR4 group showed significant upregulation, whereas the remaining 50% showed significant downregulation. Of these, seven lipid metabolites were phosphatidylethanolamine (PE), and 71% of the PE lipid metabolites showed significant upregulation (Fig. 6B). This analysis was conducted using the top 20 lipid metabolites in VIP abundance. We performed a total of the first 120 lipid substances for clustering score to further observe the changes in the relative content of lipid metabolites in the two groups. The results showed that the majority of lipid metabolites belonged to GP and GL, and the majority of these lipid metabolites, including GL, SP, and FA, showed downregulation in the BR4 group (Fig. 6C). To better understand the KEGG pathway of differential lipids in GP and GL, we conducted a cluster analysis (Fig. 6D and E). These findings indicated that the majority of lipid molecules in the KEGG pathway belonged to PC and DG, and that the majority of PCs and all of the DGs were reduced in the BR4 group. Finally, we performed KEGG pathway enrichment analysis for different lipid metabolites, the most significantly enriched pathways were metabolic pathways, followed by glycerophospholipid metabolism and retrograde endocannabinoid signaling, while arachidonic acid metabolism, amoebiasis, linoleic acid metabolism, α-linolenic acid metabolism, glycine, serine and threonine metabolism, systemic lupus erythematosus, si sarcoma-associated herpesvirus infection, and leishmaniasis were also among the significantly enriched pathways.

### Spearman correlation analysis

**Fig 7:**
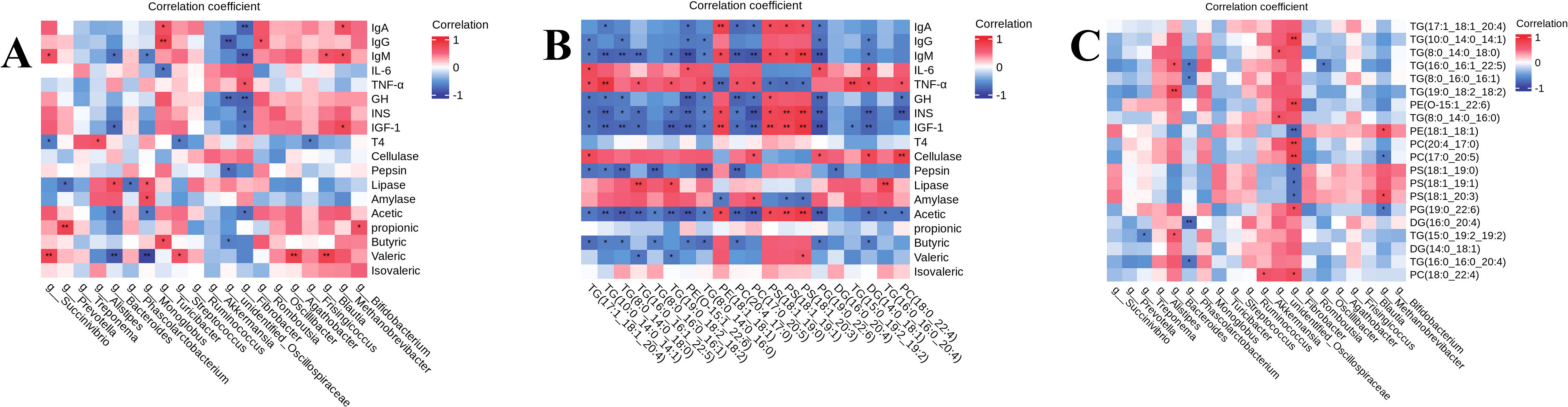
Spearman correlation analysis: (A) correlation diagram of intestinal microorganisms with immune indexes, serum hormones, enzyme activity, and VFAS. (B) The correlation between lipid metabolites and immune indexes, serum hormones, enzyme activity and VFAS. (C) Correlation between intestinal microorganisms and lipid metabolites.

The current study used Spearman’s analysis to examine the relationships between lipid metabolites and gut microbes of the top 20 with serum hormones, immune markers, intestinal enzyme activities, and VFAs, as well as with the top 20 microorganism correlations, to investigate the interactions between lipid metabolism, gut microbes, and serum immune markers, serum hormones, intestinal enzyme activities, and VFAs. The results showed that there were also significant correlations between the gut microbiota and immune markers, serum hormones, intestinal enzyme activities, and VFAS (Fig. 7A) (*P* < 0.05). For example, *Bacteroides* was significantly negatively correlated with IgM, IGF-1, acetic and valeric, and positively correlated with lipase; *Monoglobus* was significantly positively correlated with lipase and amylase, and negatively correlated with acetic and valeric; and *Fibrobacter was negatively correlated with IgA, IgM, GH, INS and IGF-1, but positively correlated with TNF-α.* Whereas lipid metabolites PS(18:1_19:0), PS(18:1_19:1), and PS(18:1_20:3) were significantly positively correlated with IgA, IgM, GH, INS, IGF-1, acetic and butyric, while significantly negatively correlated with IL-6, TNF-α, and amylase. The majority of the lipid metabolites were significantly negatively correlated with IgA, IgM, GH, IGF-1, acetic, and butyric and significantly positively correlated with TNF-α. There were also significant correlations between gut microorganisms and muscle lipid metabolites, such as significant positive correlations between *Bacteroides* and TG (16:0_16:1_22:5), TG (19:0_18:2_18:2) and TG (15:0_19:2_19:2), *Phascolarctobacterium* and TG (16:0 _16:1_22:5), TG (8:0_16:0_16:1), DG (16:0_20:4) and TG (16:0_16:0_20:4) were significantly negatively correlated; *Fibrobacter* was significantly negatively correlated with PE (18:1_18:1), PS (18:1_19:0), PS (18:1_19:1), PS (18:1_19:1), and PS (18:1 _20:3) were significantly negatively correlated with TG (10:0_14:0_14:1), PE (O-15:1_22:6), PC (20:4_17:0), PC (17:0_20:5), PG (19:0_22:6), and PC (18:0_22:4).

## DISCUSSION

Modern sheep husbandry aims to improve meat quality and animal performance in response to the growing demand for sheep meat. Herbs as feed additives have been shown in an increasing number of studies to support animal health, growth, and immunological functions (21, 22). However, it is still unclear if using herbal by-products will increase the quality of the meat or not. Studies have demonstrated that controlling the gut microbiota can enhance the growth of cattle and poultry, improve immunity and meat quality, and lower the cost of raising livestock. The intestinal tract is the largest immune organ and an important place for digestion and absorption of feed nutrients (23–26). Thus, there is a close relationship between the quality of animal products, growth and immune function, and host health and gut bacteria. Our research revealed that BR influenced muscle lipid metabolism through the regulation of gut bacteria, growth, and immune function, thereby enhancing meat quality. It also enhanced SFS growth and immunological function by controlling SFS gut microbes.

Animal growth performance is influenced by breed, age, sickness, nutrition, and environment. The primary growth rate regulators are thought to be GH, INS, IGF-1, and T4. They control the metabolism of proteins, carbohydrates, and fats, which influence the growth rate and body weight of the animal organism and positively correspond with the animal’s growth performance (27, 28). In the present study, a significant rise in the average daily weight gain was observed when BR was added to the diet at greater doses. These findings were consistent with earlier research showing that GH, INS, and IGF-1 serum levels were significantly higher (7–9). This suggested that BR could enhance SFS growth performance, with a 4% BR addition having the greatest impact. According to Pagania et al. (29) enhancing the gut microbiota may increase the release of growth hormone (GH) from the animal body, which would aid in the promotion of animal growth. Zhu et al. (30) discovered that by preventing the growth of harmful bacteria, the intestinal tract could better absorb and digest nutrients. In addition, the nutrients could be metabolized by the microbiota metabolism products, which would promote the synthesis and secretion of GH and IGF-1 and affect the growth and quality of meat in broilers. In this study, we discovered that IGF-1 was negatively connected to most TG and PC lipid metabolites, while GH, INS, and IGF-1 were significantly negatively connected to the gut microbiota of *Fibrobacter* and positively correlated with lipid metabolites like PS and PE. *Fibrobacter* was also significantly negatively correlated with PS and significantly positively correlated with PC. These results suggest that the decrease in *Fibrobacter* colonization is accompanied by increased levels of GH, INS, and IGF-1, while the lipid metabolites PS, TG, and PC are all affected. A genus of bacteria called *Fibrobacter*, which is typically present in ruminants’ digestive tracts, has a strong capacity to break down cellulose. It can also activate cellulase and take part in the body’s energy metabolism (31). PS, PC, and PG are all phospholipids, which are good antioxidants that improve metabolism and make meat more tender by promoting the deposition of intramuscular fat. They also interact with proteins and have an impact on the immunomodulation of the animal body (32, 33). Although *Fibrobacter* microbiota decreased in the intestinal tract of the BR4 group, its *Prevotella* microbiota increased, which can fully degrade crude fiber in the intestinal tract, enabling the animal organs to fully utilize starch, monosaccharides, and non-cellulosic polysaccharides in the feed (34, 35). This suggests that BR modulates the population of fibrous bacterial microbiota in the GI tract, thereby increasing serum levels of GH, INS, and IGF-1, and further regulating the metabolism of lipid molecules such as PS, PC, and PG. This improves muscle tenderness and meat quality.

Serum immune indicators can reflect the immune level of the animal organism. IgA, IgG, and IgM participate in the immune regulation of the animal organism and are important immunoglobulins in the animal organism, the higher their concentration in the serum, the stronger the immunity of the animal organism (36). Pro-inflammatory cytokines such as TNF-α and IL-6 cause inflammation in animals and promote disease progression when serum levels of these cytokines are elevated (37). Tang et al. discovered that BR could considerably boost the chicken’s immunity by enhancing the immunological signaling pathway, removing immune organ edema, and lowering the amount of IFN-α in immune organs (38). In the current study, TNF-α and IL-6 steadily dropped while the levels of IgG and IgM in SFS serum increased with the increase in BR given to the diet. According to the aforementioned findings, it is possible that adding BR to the diet can strengthen SFS’s immunity; the greatest outcomes were obtained when adding 4% of BR to the diet. Intestinal microbes, the largest immune organ, contribute significantly to immunomodulation by supplying metabolites and preserving metabolic processes (39). Research reports gut microbiota is involved in the regulation of immune and lipid metabolism in animal bodies. By increasing the beneficial gut microbiota, it can improve the immunity of animal bodies and improve meat quality (40, 41). While enhancing the quality of meat has received a lot of interest, the mechanism underlying the influence of the microbiota on immune regulation and meat quality has not been thoroughly studied. In the current study, we found that IgA, IgG, and IgM were significantly negatively connected to *Fibrobacter*, significantly positively connected to *Methanobrevibacter* and *Turicibacter*, significantly positively correlated with the lipid metabolites PS and PE, and significantly negatively correlated with TG and PC, whereas *Methanobrevibacter* and *Turicibacter* gut microbiota were not significantly correlated with lipid metabolites. *Methanobrevibacter* can produce butyric acid, which is beneficial to the health of the intestinal epithelium, by influencing the fermentation and metabolic products of intestinal bacteria and by affecting energy metabolism, whereas *Turicibacter* is an important microorganism involved in host adiposity regulation, with the ability to protect intestinal health and promote nutrient absorption and metabolism, as well as being involved in host fatty acid metabolism (42–44). TG is closely related to animal health; an excess of TG lipid substance in tissues causes adipocyte hypertrophy and hyperplasia, while lower TG metabolic levels are more favorable to animal health (45, 46). Common lipid metabolites PE, PC, PS, and TG are closely associated with meat color and tenderness. The concentration of PE and PC is positively connected to a* and negatively connected to b* (47). In the current study, TG was reduced, PC and PE lipid metabolites were elevated, and SFS immunological function was enhanced. Based on these findings, it is possible to hypothesize that BR strengthens SFS’s defense against infection by controlling the gut microbiota of *Fibrobacter*, *Methanobrevibacter*, and *Turicibacter* in SFS. This, in turn, influences the metabolism of PE, PC, PS, and TG as well as other lipids, improving the health of the animal and the color of its meat. More intriguingly, lipid metabolic pathways were enriched in this study primarily for retrograde endocannabinoid signaling, phospholipid metabolism, and glycophospholipid metabolism, which are involved in immune regulation and lipid metabolism in animals. Any disruption of these pathways can result in fatty and hepatic lesions as well as a decrease in immune performance (48–50). Myers et al. (51) found that retrograde endocannabinoid signaling has a unique regulatory effect on lipolysis, and stimulation of lipolysis increases retrograde endocannabinoid signaling. Phospholipid metabolism and glycophospholipid metabolism are directly involved in the regulation of lipid metabolism disorders. Normal phospholipid metabolism and glycophospholipid metabolism can enhance lipid deposition in animal bodies (52, 53). Second, linoleic acid metabolism, alpha-linolenic acid metabolism, glycine, serine, and threonine metabolism can improve fat deposition, prevent lipid metabolism disorders, inhibit the harmful gut microbiota, and promote animal health (54–56). Our hypothesis was that including BR in diets would control the pathways involved in lipid metabolism, consequently boosting SFS’s immunological qualities and gut microbiota, which would further influence lipid metabolism and improve the quality of meat.

The digestive system is a crucial location for the digestion and absorption of nutrients, and the gut microbiota helps to maintain the health of the host through symbiosis and mutual support with the host (57). Therefore, in this experiment, the effect of BR on the gut microbiota of lambs was analyzed using 16sRNA. The results showed that there was a significant difference between the bacterial microbiota of the CON and BR4 groups., with *Firmicutes*, *Bacteroidota*, *Succinivibrio*, *Treponema*, *Bacteroides*, and *Prevotella* being the most abundant microbiota in the gut, and all of these microbiota belong to the group of beneficial microbiota, which are involved in nutritional health through the regulation of enzyme activity and VFAs, which are involved in the digestion, absorption, and metabolic regulation of nutrients (58–61). *Fibrobacterota, Cyanobacteria, Anaerovorax, Flavonifractor*, and *Desulfovibrio* were found to be significantly less prevalent in the BR4 group during the differential analysis of bacterial microbiota. These bacteria affect immune regulation, produce harmful substances, and cause intestinal metabolic disorders that negatively impact animal organisms (62, 63). The abundance of gut microbiota such as *Ralstonia*, *Mogibacterium*, *Roseburia*, *Atopobium,* and *Oscillospira* was significantly higher in the BR4 group. These microbial ginsengs can prevent the formation of dangerous bacteria, regulate the metabolism of fatty acids and purines, and aid in the absorption and utilization of nutrients (64, 65). According to our hypothesis, adding BR to the diet could raise the relative abundance of healthy gut microbiota in SFS, preventing gut microbiota disorders and preserving the health of the lambs while also controlling the quality of the meat due to the effect of gut microbiota on lipid metabolism. The purpose of this investigation was to correlate the muscle quality using tissue sectioning and LC-MS/MS techniques to better examine the impact of BR on the meat quality of SFS. The muscle fibers in the BR4 group had a small diameter and a higher density, according to the tissue section data. It has been demonstrated that a muscle’s density and relatively high intramuscular fat content increase with a muscle’s smaller diameter. This lowers shear force, enhances meat tenderness, and enhances overall meat quality (66). It is demonstrable that BR can raise the quality of SFS meat. One of the key elements influencing the quality of meat is its intramuscular fat content. A high level of muscle lipid can make the meat more tender, and the fatty acids that are released during the breakdown of intramuscular lipid substances can influence the flavor of the meat as well as its nutritional value (67). According to the lipid metabolism data, the BR4 group had much higher levels of lipid metabolism molecules such as PE, PS, and PG and significantly lower levels of PC and DG. Research indicates that while PC and DG harmonize with muscle nutrition and promote fat deposition, PE and PS enhance meat color and tenderness (47, 48). According to the above findings, it is possible that including BR in the diet will enhance SFS, which will raise the density of muscle fibers, as well as improve meat color, tenderness, and nutrition by controlling lipid metabolites including PE, PS, PG, PC, and DG. Enhancing gut microbiota has been shown in studies to influence intestine VFAs and enzyme activity levels, which, in turn, impacts lipid metabolism and enhances meat quality (68, 69). It was discovered that in this experiment, there was a substantial positive correlation between PS and PE lipid metabolites and a significant negative correlation between TG and DG lipid metabolites and pepsin, amylase, acetic, butyric, and valeric. While gut microbiota *Bacteroides* and *Monoglobus* showed a significant positive correlation with lipase and amylase, and a significant negative correlation with acetic and valeric. A large number of studies have shown that intestinal active enzymes can be involved in nutrient absorption and transportation, in which the increased activity of pepsin and amylase can improve the growth performance and carcass traits of animals and reduce the deposition of abdominal fat. Whereas VFAs are involved in the regulation of gut microbiota and can inhibit the harmful intestinal microbiota when the microorganisms decompose in large quantities to produce acetic, propionic, and butyric, and the flavor of meat will be improved accordingly (70–72). In this investigation, lipid metabolites TG and DG were decreased, but lipid metabolites PS and PE were elevated. Pepsin, acetic acid, and butyric acid levels were considerably enhanced in the BR4 group. The addition of BR to the diet may have an impact on the synthesis of VFAs, including acetic, propionic, and butyric, as well as increase pepsin activity by controlling the bacterial microbiota in the intestine. This may have an impact on the muscle’s lipid metabolism and enhance the color, tenderness, flavor, and nutritional content of the meat. In the current study, TG was the lipid metabolite with which *Bacteroides* exhibited a significant positive correlation, TG and *Phascolarctobacterium* demonstrated a significant negative correlation, TG and *Fibrobacter* demonstrated a significant negative correlation, and PC and PG demonstrated a significant positive association. *Bacteroidota, Phascolarctobacterium,* and *Fibrobacter* are the main microbiota that produce VFAs, which are catabolized in the intestine to produce large amounts of acetic, propionic, and butyric, which can improve feed utilization (73, 74). As a member of the phylum *Firmicutes*, *Monoglobus* is a pectin-degrading microbiota that can break down a range of galactans and pectins to produce a number of compounds that can be used by other bacteria for sugar degradation (75). In addition to VFAs, it is possible that gut microbiota like Bacteroidota, Phascolarctobacterium, and Fibrobacter directly affect lipid metabolism.

### Conclusions

In conclusion, dietary supplementation of BR can improve the growth and immune performance of SFS. Second, the improvement of gut microbiota will regulate VFAs and enzyme activities, further affect lipid metabolism, and improve meat quality. Key microorganisms involved in lipid regulation include *Fibrobacter, Bacteroides,* and *Phascolarctobacterium.* The key lipid metabolites regulated by gut microbiota and affecting meat quality include TG, DG, PE, PS, PC, and PG. Furthermore, immune modulation was regulated by the lipid metabolic pathways in the BR4 group, including the glycerophospholipid metabolism, retrograde endocannabinoid signaling, and the metabolic pathways. Research has demonstrated that herbal byproducts can improve the growth of livestock and poultry and the quality of livestock products, which provides a new perspective of the potential of herbal by-products as feed additives.

## Data Availability

All data used in this study are available upon request from the corresponding or first author. In addition, the datasetdata set created specifically for this study has been deposited in the NCBI Sequence Read Archive, which can be found under the BioProject identifier PRJNA1104792.
